# Practice patterns of endoscopic ultrasound-guided tissue acquisition of solid pancreatic masses: an international survey study

**DOI:** 10.1007/s00464-026-13100-0

**Published:** 2026-07-14

**Authors:** Samuel Tanner, Ricardo Marin, Somashekar G. Krishna, Alberto Espino, Naveen Arya, Yen-I Chen, Vinay Dhir, Antonio Facciorusso, Nauzer Forbes, Anand V. Sahai, Anthony Teoh, Sachin Wani, C. Mel Wilcox, Veeral M. Oza, Truptesh Kothari, Sergio A. Sánchez-Luna, Jorge D. Machicado

**Affiliations:** 1 Division of Gastroenterology and Hepatology, University of Michigan, Ann Arbor, MI USA; 2 Division of Gastroenterology and Hepatology, University of Louisville, Louisville, KY USA; 3 Division of Gastroenterology, Hepatology & Nutrition, The Ohio State University Wexner Medical Center, Columbus, OH USA; 4 Department of Gastroenterology, School of Medicine, Pontificia Universidad Católica de Chile, Santiago, Chile; 5 Division of Gastroenterology, Oakville Trafalgar Memorial Hospital, Oakville, ON Canada; 6 Division of Gastroenterology and Hepatology, McGill University Health Centre, McGill University, Montreal, QC Canada; 7 Institute of Digestive and Liver Care, SL Raheja Hospital, Mumbai, India; 8 Gastroenterology Unit, Department of Experimental Medicine, Università del Salento, Lecce, Italy; 9 Clinical Effectiveness Research Group, University of Oslo, Oslo, Norway; 10 Department of Community Health Sciences, University of Calgary, Calgary, AB Canada; 11 Division of Gastroenterology and Hepatology, Department of Medicine, University of Calgary, Calgary, AB Canada; 12 Department of Medicine, Centre Hospitalier de L’Université de Montréal, Montreal, QC Canada; 13 Hong Kong Sanatorium & Hospital, Hong Kong SAR, China; 14 Division of Gastroenterology and Hepatology, University of Colorado Anschutz Medical Campus, Aurora, CO USA; 15 Digestive Health Institute, Orlando Health, Orlando, FL USA; 16 Bon Secours Mercy Health, Gastroenterology and Liver Disease, Greenville, SC USA; 17 Division of Gastroenterology and Hepatology, University of Rochester Medical Center, Rochester, NY USA; 18 Instituto Mexicano de Cirugía Endoscópica y Bariátrica (IMCEB), Guadalajara, México; 19 Division of Gastroenterology & Hepatology, Department of Internal Medicine, Basil I. Hirschowitz Endoscopic Center of Excellence, The University of Alabama at Birmingham Heersink School of Medicine, Birmingham, AL USA

**Keywords:** Pancreatic cancer, Pancreas mass, Fine needle biopsy, Needle aspiration

## Abstract

**Background:**

Advancements in endoscopic ultrasound-guided tissue acquisition (EUS-TA) for solid pancreatic masses have prompted updates in society guideline recommendations. However, the extent to which these recommendations have been adopted in clinical practice remains unclear. Thus, we sought to characterize current practice patterns and beliefs regarding EUS-TA for solid pancreatic masses in a large, international cohort of endosonographers.

**Methods:**

We developed and distributed an electronic survey to 1,281 endosonographers worldwide between September and November 2024. The survey aimed to evaluate EUS-TA practices across six domains: A) needle selection, B) sampling technique, C) method guiding the number of passes, D) specimen handling, E) quality indicator (QI) monitoring, and F) factors influencing decision-making.

**Results:**

The response rate was 30% (*n* = 376). Most endosonographers often or always use fine needle biopsy (FNB) needles (90%) of 22-gauge (85%) and Franseen design (53%). The majority use a fanning technique (79%) and perform ≤ 10 actuations (60%). The number of FNB passes is mostly guided by visual on-site evaluation (60%). Advanced EUS imaging utilization was low (EUS elastography: 14%; contrast-enhanced EUS: 8%). Institutional QI monitoring was reported by 51% of endosonographers. Compared to other regions, endosonographers in North America are more likely to use rapid on-site evaluation, but less likely to use advanced EUS imaging modalities or have QI monitoring (all *p* < 0.001).

**Conclusion:**

There are significant global variations in EUS-TA practices for solid pancreatic masses. These survey data provide a benchmark to guide standardization, inform quality monitoring, and support evolving technologies to optimize diagnostic outcomes and patient care worldwide.

**Supplementary Information:**

The online version contains supplementary material available at https://doi.org/10.1007/s00464-026-13100-0.

Numerous high-quality studies have emerged over the past decade comparing critical aspects of endoscopic ultrasound-guided tissue acquisition (EUS-TA) for solid pancreatic masses including needle selection, sampling technique, and specimen handling [1–5]. In response, professional societies including the American Society for Gastrointestinal Endoscopy (ASGE), European Society of Gastrointestinal Endoscopy (ESGE), and Asian Endoscopic Ultrasound Group (AEG) have issued updated evidence-based guidelines for EUS-TA for solid pancreatic masses [6–8]. These guidelines generally agree that i) fine needle biopsy (FNB) with end-cutting tip needles are preferred over fine needle aspiration (FNA) or first generation FNB needles, ii) routine rapid on-site evaluation (ROSE) is not required when using FNB needles, and iii) no specific suction technique is favored. However, notable differences exist, particularly in recommendations regarding macroscopic on-site evaluation (MOSE), needle movement, and preferred needle size.

Despite these advances, only a few studies have investigated how endosonographers perform EUS-TA for solid pancreatic masses in real-world clinical settings [9–11]. These survey studies, however, were conducted before the widespread availability of contemporary FNB needles and were limited by small sample sizes, a restricted population of endosonographers, and a focus on a narrow subset of practice parameters such as needle type, needle size, and methodology used to guide the number of passes. Other important aspects of EUS-TA for solid pancreatic masses, including the application of advanced imaging modalities (e.g., EUS elastography, contrast-enhanced EUS), use of quality indicators (QI), and factors influencing decision-making, have not been previously explored. Given these gaps, a global assessment of current practice patterns of EUS-TA for solid pancreatic masses is essential to understand guideline adoption and areas of continued variability, especially in an era where FNB has become the primary recommended method. Therefore, we conducted an international survey to assess contemporary practices of endosonographers performing EUS-TA for solid pancreatic masses and to evaluate how geographic location, experience, and procedural volume influence these patterns.

## Methods

### Study design

This was an international survey study conducted between September and November 2024. The survey instrument was approved by the University of Michigan Institutional Review Board (HUM00260673).

### Survey instrument development

A list of potential questions was developed following a comprehensive literature review and focused discussions with leaders in the field of EUS-TA. Content validity and pilot testing was then performed among 15 international EUS-TA experts from North America, Europe, Asia, and Latin America using a mixed-method approach. The final survey included 23 questions (Supplementary Appendix A) focusing on six domains of EUS-TA for solid pancreatic masses: A) needle selection, B) sampling technique, C) method to guide the number of passes, D) specimen handling, E) local QI monitoring, and F) factors affecting decision-making. The questions were presented in English and were not translated.

### Study population

A directory of international endosonographers and corresponding e-mail addresses was developed using different methods. In the US, a contact list was developed using the ASGE advanced endoscopy fellowship website, ASGE active membership, and personal contacts. In other regions, endosonographers were identified through a comprehensive approach that included direct collaboration with regional EUS leaders, review of participant lists from international EUS conferences, and referrals from recognized EUS experts within the global community.

### Survey distribution

The final survey was uploaded in REDCap (Research Electronic Data Capture), a commonly used electronic data capture tool, and was hosted at the University of Michigan [12, 13]. The survey was electronically distributed to potential participants between September and November 2024 via individual e-mails that included a link to the survey. A total of four e-mail reminders were sent to potential participants who did not complete the survey. All data were deidentified and anonymously tabulated in the REDCap software.

### Study variables

Baseline demographics were collected, including geographic location, practice setting, years of independent practice, average annual number of EUS procedures, and participation in training of future endosonographers. Respondents were asked how often they use FNB needles and FNA needles for EUS-TA from a scale of 0–100%. If respondents reported using FNB needles for EUS-TA for solid pancreatic masses, they were asked questions pertaining to domains A-D. *Domain A* assessed the utilization of different FNB needle types and needle calibers on a scale from 0 to 100%. *Domain B* evaluated needle movement, sampling technique of the first and subsequent passes, and number of actuations. *Domain C* estimated the methodology used to determine the number of passes (ROSE, MOSE, visual on-site evaluation [VOSE], or pre-determined number of passes) on a scale of 0–100%. MOSE was defined as an aggregated specimen length of macroscopically visible core measuring ~ 10 mm, while VOSE was defined as the visual presence of worm-like whitish or yellowish material. *Domain D* assessed the technique to express the tissue out of the needle and the preferred medium to send the specimen for evaluation. If respondents reported FNA needle use for > 20% of EUS-TAs, similar questions were asked about their practices using FNA needles (*n* = 64, not reported). Regardless of the needle used, all respondents were asked on how frequently advanced EUS imaging modalities (EUS elastography, contrast-enhanced EUS) are used to guide sampling. *Domain E* was assessed in all respondents to explore if institutions monitor quality indicators related to EUS-TA and the type of measures that are monitored. Finally, *domain F* involved ranking on a 5-point Likert scale (most important: 1; to least important: 5) the factors that are considered when selecting across EUS needles, sampling techniques, and methods to guide the number of passes.

### Statistical analysis

To be included in the analysis, participants had to self-report that they perform EUS and had to complete at least one question in any of the 6 survey domains. Descriptive statistics were reported as proportions for categorical data and as mean ± standard deviation for continuous data. Responses where participants were asked to rate their use patterns from a scale of 0 to 100% were converted to ordinal variables with 0–10% categorized as never, 11–40% as rarely, 41–60% as sometimes, 61–90% as often, and 91–100% as always. Responses to 5-point Likert scales were treated as continuous variables.

Preplanned subgroup analyses were conducted based on practice location (North America, Latin America, Asia, or Europe), volume of yearly EUS-TAs for solid pancreatic masses (≤ 200: low volume vs. > 200: high volume), and years of experience in independent practice (≤ 10: early career vs. > 10: experienced). Responses from Australia (*n* = 9) were reported together with Asia given their small number. Additionally, Central America (*n* = 21) and South America (*n* = 26) were reported together as Latin America. For subgroup analyses, responses where participants were asked to rate their use patterns from a scale of 0 to 100% were dichotomized with ‘preferred’ being defined as using a particular item or technique for > 60% of the time. Univariate comparisons were performed using the chi-squared test for categorical data and using the Mann–Whitney U or Kruskal–Wallis tests for continuous data. Statistical significance was defined as a two-sided *p* value of < 0.05. Statistical analysis was performed using SPSS Statistics v29.0.2.0 (IBM, Armonk, New York, USA).

## Results

### Demographic characteristics

Out of 1,271 invited participants, a total of 376 endosonographers provided valid survey responses (response rate of 30%). Of these, 330 (86%) completed the entire survey, while the rest provided partial responses. Nearly half of respondents practiced in the United States (46%), while the rest practiced in Asia (17%), Europe (16%), Latin America (13%), Canada (7%), and Australia (2%). The majority practiced in a university hospital (67%), had > 5 years of EUS experience (76%), performed > 200 EUS-TA procedures a year (71%), and participated in the training of future endosonographers (76%, Table 1).

**Table 1:** Demographics of participating endosonographers

Variable (*n*)	*n* (%)
Practice location (*n* = 374)	
United States	170 (46)
Canada	26 (7)
Central America	21 (6)
South America	26 (7)
Europe	57 (15)
Asia	65 (17)
Australia	9 (2)
Type of practice (n = 375)^1^	
University hospital	252 (67)
Private practice	84 (22)
Community practice	70 (19)
Veteran affairs hospital	12 (3)
Other	11 (3)
Years of independent practice (*n* = 374)	
< 5	88 (24)
5 to 10	96 (26)
11 to 20	98 (26)
> 20	92 (25)
EUS-TA procedures per year (*n* = 372)	
< 100	27 (7)
101 to 200	82 (22)
201 to 300	96 (26)
301 to 400	61 (16)
> 400	106 (29)
Participate in training of future endosonographers (*n* = 375)	
Yes	283 (76)
No	92 (25)

*EUS-TA* Endoscopic ultrasound-guided tissue acquisition

^1^ Respondents were allowed to select more than one option

### Overall survey results

#### Needle selection

**Fig. 1:**
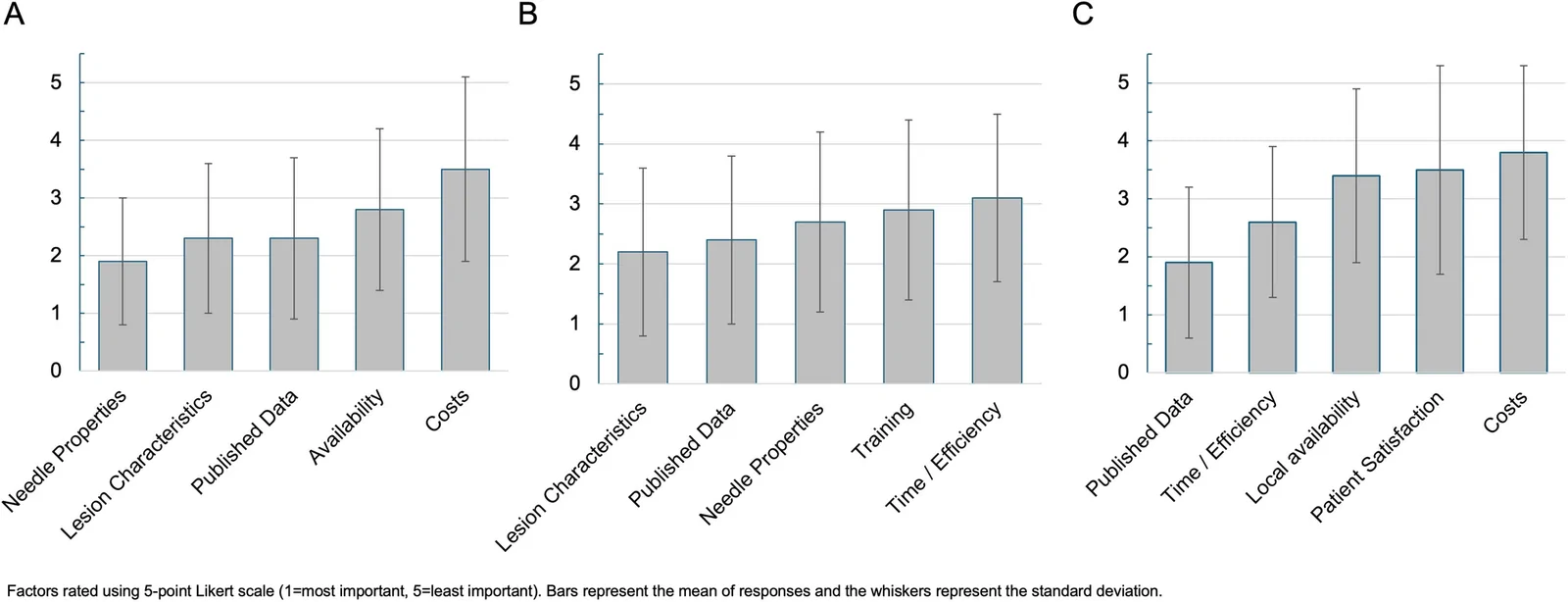
Importance of different factors in determining **A** needle selection, **B** sampling technique, and **C** method to determine the total number of passes performed

A preference for FNB needles was evident among respondents, with 90% reporting they always or often use FNB for EUS-TA of solid pancreatic masses, while 76% indicated they never use FNA needles for these procedures (Table 2). When using FNB needles, most endosonographers always or often used a 22-G needle size (85%). A majority (53%) of endosonographers always or often selected a needle with a Franseen design, while 23% always or often selected a needle with a Fork-Tip design. When selecting a needle for EUS-TA, endosonographers placed the highest importance on needle properties including its sharpness and maneuverability (mean Likert score: 1.9 ± 1.1), followed by lesion characteristics including location and size (2.3 ± 1.3), and supporting published data related to the specific needle (2.3 ± 1.4, Fig. 1A).

**Table 2:** Practice patterns on selection of EUS needles

Variable (*n*)	Always	Often	Sometimes	Rarely	Never
Preferred needle type					
FNB needle (*n* = 376)	259 (69)	80 (21)	10 (3)	12 (3)	15 (4)
FNA needle (*n* = 375)	19 (5)	16 (4)	14 (4)	43 (12)	283 (76)
Needle gauge (*n* = 362)					
25-G	4 (1)	19 (5)	10 (3)	46 (13)	283 (78)
22-G	164 (45)	145 (40)	20 (6)	18 (5)	15 (4)
21-G	1 (0)	1 (0)	3 (1)	0 (0)	357 (99)
20-G	1 (0)	4 (1)	7 (2)	5 (1)	345 (95)
19-G	4 (1)	4 (1)	6 (2)	37 (10)	311 (86)
Needle design					
SharkCore (Fork-Tip) (*n* = 363)	37 (10)	46 (13)	21 (6)	29 (8)	230 (63)
Acquire (Franseen) (*n* = 365)	78 (21)	78 (21)	36 (10)	45 (12)	128 (35)
Acquire S (Franseen) (*n* = 365)	14 (4)	16 (4)	14 (4)	20 (6)	301 (83)
SonoTip TopGain (Franseen) (*n* = 365)	4 (1)	2 (1)	2 (1)	6 (2)	351 (96)
EZ Shot 3 plus (Menghini) (*n* = 365)	4 (1)	12 (3)	8 (2)	20 (6)	321 (88)
EchoTip ProCore (Reverse-bevel) (*n* = 365)	5 (1)	4 (1)	10 (3)	17 (5)	329 (90)
EchoTip AcuCore (Franseen) (*n* = 363)	1 (0)	3 (1)	3 (1)	3 (1)	353 (97)
Trident (Tri-Tip) (*n* = 365)	5 (1)	7 (2)	6 (2)	10 (3)	337 (92)
Utilization of advanced EUS imaging					
EUS elastography (*n* = 342)	22 (6)	27 (8)	29 (9)	37 (11)	227 (66)
Contrast-enhanced EUS (*n* = 341)	10 (3)	16 (5)	18 (5)	22 (7)	275 (81)

*EUS* Endoscopic ultrasound, *FNB* Fine needle biopsy, *FNA* Fine needle aspiration

SharkCore, Medtronic, Minneapolis, USA; Acquire, Boston Scientific, Marlborough, USA; Acquire S, Boston Scientific, Marlborough, USA; SonoTip TopGain, Medi-Globe, Germany; EZ Shot 3 Plus, Olympus, Tokyo, Japan; EchoTip ProCore; Cook Medical, Indiana, USA; EchoTip AcuCore, Cook Medical, Indiana, USA; Trident, Microtech, Nanjing, China

#### Sampling technique

When using FNB needles, 79% of endosonographers preferred a fanning technique (Table 3). For the first FNB pass, the most common suction techniques were a stylet slow-pull alone (49%) and stylet slow pull followed by wet or dry suction (27%). For subsequent FNB passes, only 15% often or always changed their initial sampling approach. For each FNB pass, most endosonographers reported performing 6–10 actuations (41%) followed by 11–15 actuations (22%) and ≤ 5 actuations (19%). When selecting the sampling technique, endosonographers placed the highest importance on lesion characteristics (2.2 ± 1.4), published data (2.4 ± 1.4), and needle characteristics (2.7 ± 1.5) (Fig. 1B). A small subset of endosonographers always or often used EUS elastography (14%) or contrast-enhanced EUS (8%) to guide the area to sample in a pancreatic mass.

**Table 3:** Practice patterns on sampling technique, specimen handling, and quality monitoring

Variable	*N* (%)
Needle movement (*n* = 353)	
Fanning	278 (79)
To-and-Fro	57 (16)
Other/not applicable	18 (5)
Initial suction technique (*n* = 352)	
Stylet slow-pull alone	171 (49)
Stylet slow-pull followed by dry or wet suction	94 (27)
Dry suction alone^1^	40 (11)
Plain needle alone without slow-pull or suction	19 (5)
Wet suction alone^2^	18 (5)
Other	10 (3)
Change in subsequent sampling technique (*n* = 352)	
Always	17 (5)
Often	23 (10)
Sometimes	114 (32)
Rarely	143 (41)
Never	44 (13)
Number of actuations per pass (*n* = 350)	
≤ 5	66 (19)
6 to 10	142 (41)
11 to 15	77 (22)
16 to 20	37 (11)
> 20	20 (6)
EUS elastography utilization (*n* = 342)	
Always	22 (6)
Often	27 (8)
Sometimes	29 (9)
Rarely	37 (11)
Never	227 (66)
Contrast-enhanced EUS utilization (*n* = 341)	
Always	10 (3)
Often	16 (5)
Sometimes	18 (5)
Rarely	22 (7)
Never	275 (81)
Sample expression technique (*n* = 344)	
Stylet	107 (31)
Air flush	22 (6)
Saline flush	22 (6)
Stylet and air flush	74 (22)
Stylet and saline flush	118 (34)
Preferred sample medium (*n* = 340)	
Formalin 10%	274 (81)
CytoLyt®	47 (14)
ThinPrep®	6 (2)
Saline	7 (2)
Alcohol	2 (1)
Institutional QI monitoring (*n* = 330)	168 (51)
Type of monitored QI metric (*n* = 168)	
Tissue adequacy	117 (70)
Diagnostic accuracy	151 (90)
Adverse events	120 (71)
Non-diagnostic samples	119 (71)
Appropriate photo-documentation/measurements	59 (35)

*EUS* endoscopic ultrasound, *QI* quality indicator

^1^ Defined as applying negative pressure with a syringe. ^2^ Defined by pre-flushing the needle with saline or sterile water, in addition to applying negative pressure with a syringe

#### Methods to guide the number of passes

VOSE was the most common method to guide the number of FNB needle passes, being always or often used by 60% of endosonographers. Less commonly, endosonographers always or often used ROSE (25%), MOSE (21%) or a predefined number of passes (15%; Table 4). When a predefined number of passes was used, endosonographers reported usually performing two or three passes. Published data (1.9 ± 1.3) and time efficiency (2.6 ± 1.3) were the most important considerations in selecting how to determine the number of FNB passes (Fig. 1C). Endosonographers who used ROSE reported that a preliminary diagnostic interpretation was always or often provided in 61% of cases. These endosonographers used this preliminary diagnostic interpretation to support their diagnostic confidence (2.1 ± 1.0), guide discussions with patients immediately following the procedure (2.3 ± 1.3), and expedite referrals to specialists (2.3 ± 1.2, Supplementary Fig. 1).

**Table 4:** Practice patterns on the methods to guide the number of FNB passes

Variable	Always	Often	Sometimes	Rarely	Never
Method to determine # of passes (*n* = 339)					
VOSE^1^	151 (45)	52 (15)	42 (12)	22 (7)	72 (21)
MOSE^2^	55 (16)	18 (5)	32 (9)	36 (11)	198 (58)
ROSE	42 (12)	45 (13)	25 (7)	31 (9)	196 (58)
Predefined	31 (9)	20 (6)	34 (10)	43 (13)	211 (62)
Predefined passes performed					
1 pass (*n* = 129)	5 (4)	7 (5)	17 (13)	47 (36)	53 (41)
2 passes (*n* = 132)	26 (20)	37 (38)	17 (13)	25 (19)	27 (21)
3 passes (*n* = 153)	38 (25)	46 (30)	36 (24)	18 (12)	15 (10)
4 passes (*n* = 138)	14 (10)	33 (24)	31 (23)	38 (28)	22 (16)
5 passes (*n* = 132)	4 (3)	14 (11)	21 (16)	48 (36)	45 (34)
6 passes (*n* = 128)	0 (0)	9 (7)	9 (7)	34 (27)	76 (59)
≥ 7 passes (*n* = 124)	4 (3)	1 (1)	1 (1)	31 (25)	87 (70)

*VOSE* Visual on-site evaluation, *MOSE* Macroscopic on-site evaluation, *ROSE* Rapid on-site cytopathology evaluation

^1^ Defined by presence of worm-like whitish or yellowish material. ^2^ Defined by an aggregated specimen length of macroscopically visible core that measures ~ 10 mm or more

#### Specimen handling

The specimens were most commonly expressed from the FNB needle using a stylet in combination with saline flush (34%) followed by stylet alone (31%) and stylet with air flush (21%, Table 3). The specimens were generally put into 10% Formalin (80%).

#### Quality monitoring

Half of respondents (51%) reported that their institutions monitored outcomes of EUS-TAs for solid pancreatic masses (Table 3). In those with institutional monitoring, measured quality metrics included diagnostic accuracy (90%), non-diagnostic samples (71%), adverse events (71%), and tissue adequacy (70%).

### Geographic variations

There was consensus among endosonographers across different continents on preferring FNB needles (86–92%) and 22-G caliber size (81–90%), although specific needle designs varied across regions (Table 5). Endosonographers in North America favored a Fork-Tip design (36% vs. 2–16% in other regions, *p* < 0.001) and in Latin America and Europe favored a Franseen design (58% and 61% versus 34–42%, *p* < 0.001). The fanning technique was preferred across all regions, although this technique was more common in Asia (89%) and Latin America (95%) than in North America (72%) or Europe (77%, *p* < 0.01). Endosonographers in North America were less likely to use EUS elastography (4% North America vs. 43% Latin America, 27% Asia, 20% Europe; *p* < 0.001) or contrast-enhanced EUS (1% North America vs. 31% Asia, 16% Europe, 5% Latin America; *p* < 0.001). While VOSE was preferred in all regions when determining the number of passes with FNB needles (55–76%), there was higher use of ROSE in North America (38% vs. 7–19% in other regions, *p* < 0.001) and MOSE in Latin America and Europe (31% and 43% vs. 13–20%; *p* < 0.001). Costs and needle availability were less important factors in needle selection for endosonographers in North America as compared to those from other regions (*p* < 0.05). Quality metrics of EUS-TA for solid pancreatic masses were less commonly monitored in North America (33%) as compared to practices in Asia (81%), Latin America (70%), and Europe (63%, *p* < 0.001).

**Table 5:** Regional variations in preferred practices for EUS-TA of solid pancreatic masses with FNB needles

	North America (*n* = 196)	Latin America (*n* = 47)	Europe (*n* = 74)	Asia (*n* = 57)	*p*-value
Needle type					
FNB needle	180/196 (92)	43/47 (91)	65/74 (88)	49/57 (86)	0.51
FNA needle	17/196 (9)	5/46 (11)	8/74 (11)	5/57 (9)	0.93
Needle gauge					
25-G	20/190 (11)	1/44 (2)	0/69 (0)	2/57 (4)	< 0.01
22-G	159/190 (85)	39/44 (87)	62/69 (90)	43/57 (81)	0.59
Needle design					
SharkCore (Fork-Tip)	68/190 (36)	1/45 (2)	5/71 (7)	9/57 (16)	< 0.001
Acquire (Franseen)	63/190 (33)	26/45 (58)	41/71 (61)	24/57 (42)	< 0.001
Acquire S (Franseen)	19/190 (10)	1/45 (2)	4/71 (6)	6/57 (14)	0.27
SonoTip TopGain (Franseen)	0/190 (0)	2/45 (4)	1/71 (3)	3/57 (5)	0.02
EZ Shot 3 plus (Menghini)	9/190 (5)	3/45 (7)	4/71 (6)	1/57 (2)	0.62
EchoTip ProCore (Reverse-bevel)	3/190 (2)	1/45 (2)	3/71 (4)	2/57 (4)	0.62
EchoTip AcuCore (Franseen)	3/190 (2)	0/45 (0)	1/71 (1)	0/57 (0)	0.66
Trident (Tri-Tip)	2/190 (1)	6/45 (13)	2/71 (4)	2/57 (4)	< 0.001
Needle movement					
Fanning	135/187 (72)	42/44 (95)	50/65 (77)	49/55 (89)	< 0.01
To-and-Fro	41/187 (22)	1/44 (2)	13/65 (20)	2/55 (4)	< 0.001
Utilization of advanced EUS imaging					
EUS elastography	7/181 (4)	17/40 (43)	13/64 (20)	15/55 (27)	< 0.001
Contrast-Enhanced EUS	1/181 (1)	2/41 (5)	8/65 (16)	15/55 (31)	< 0.001
Method to determine # of passes					
VOSE^2^	95/177 (55)	29/42 (69)	37/63 (59)	42/55 (76)	0.01
MOSE^3^	22/177 (13)	13/42 (31)	27/63 (43)	11/55 (20)	< 0.001
ROSE	66/177 (38)	7/42 (17)	11/63 (19)	3/55 (7)	< 0.001
Predefined	20/177 (11)	9/42 (21)	11/63 (17)	11/55 (20)	0.20
Institutional QI monitoring (*n* = 330)	58/175 (33)	28/40 (70)	38/60 (63)	43/53 (81)	< 0.001
Needle selection criteria (*n* = 357)^4^					
Needle properties	1.8 ± 1.0	2.1 ± 1.3	2.2 ± 1.4	1.7 ± 1.1	0.06
Lesion characteristics	2.5 ± 1.2	2.3 ± 1.6	2.2 ± 1.2	2.1 ± 1.2	0.28
Published data	2.4 ± 1.3	2.4 ± 1.6	2.4 ± 1.4	2.1 ± 1.3	0.35
Cost	3.8 ± 1.3	3.1 ± 1.5	3.2 ± 1.4	3.0 ± 1.5	< 0.001
Availability	3.0 ± 1.5	2.4 ± 1.7	3.0 ± 1.6	2.5 ± 1.7	0.04
Method to determine sampling technique (*n* = 345)^4^					
Needle properties	2.8 ± 1.5	2.6 ± 1.7	1.9 ± 1.4	3.1 ± 1.5	< 0.001
Lesion characteristics	2.3 ± 1.4	2.0 ± 1.4	2.0 ± 1.2	2.2 ± 1.5	0.08
Published data	2.6 ± 1.4	2.1 ± 1.5	2.5 ± 1.4	2.3 ± 1.4	0.08
Training	3.1 ± 1.6	2.5 ± 1.4	2.8 ± 1.4	2.6 ± 1.4	0.10
Time/Efficiency	3.1 ± 1.4	2.7 ± 1.6	3.3 ± 1.3	3.0 ± 1.4	0.09
Method to determine # of needle passes (*n* = 330)^4^					
Published data	2.1 ± 1.3	1.7 ± 1.2	1.7 ± 1.2	1.8 ± 1.3	0.10
Time	2.7 ± 1.3	2.2 ± 1.3	2.7 ± 1.1	2.7 ± 1.4	0.13
Costs	4.4 ± 1.2	3.3 ± 1.9	3.6 ± 1.5	3.3 ± 1.4	< 0.01
Availability	3.3 ± 1.8	3.4 ± 2.0	3.9 ± 1.7	3.5 ± 1.8	0.22
Patient satisfaction	3.7 ± 1.5	3.3 ± 1.7	3.4 ± 1.5	3.3 ± 1.6	0.19

*EUS-TA* Endoscopic ultrasound-guided tissue acquisition, *FNB* Fine needle biopsy, *FNA* Fine needle aspiration, *QI* Quality indicator

^1^ Defined as proportion of respondents who use item or technique in > 60% of EUS-TAs of solid pancreatic masses. ^2^ Defined by presence of worm-like whitish or yellowish material. ^3^ Defined by an aggregated specimen length of macroscopically visible core that measures ~ 10 mm or more. ^4^ Reported as mean ± standard deviation with responses to 5-point Likert scales recorded as 1 being the most important and 5 being the least important and treated as continuous variables

SharkCore, Medtronic, Minneapolis, USA; Acquire, Boston Scientific, Marlborough, USA; Acquire S, Boston Scientific, Marlborough, USA; SonoTip TopGain, Medi-Globe, Germany; EZ Shot 3 Plus, Olympus, Tokyo, Japan; EchoTip ProCore; Cook Medical, Indiana, USA; EchoTip AcuCore, Cook Medical, Indiana, USA; Trident, Microtech, Nanjing, China

### Variations by endosonographer experience and practice volume

There were no significant differences in practice patterns based on endosonographer experience (Supplementary Table 1) or endosonographers practice volume (Supplementary Table 2).

## Discussion

This international survey study captures real-world practice patterns in EUS-TA. While the adoption of evidence-based approaches for EUS-TA of solid pancreatic masses appears widespread, the finding that quality indicators are not routinely monitored across many practices is notable. This highlights an important opportunity to enhance procedural consistency and outcomes through more structured quality oversight. Although some variation in the use of adjunct tools, such as advanced imaging, was observed, this was less central to overall practice differences. The presence of regional variation in key aspects of practice underscores the need for unified guidelines and greater global collaboration to optimize outcomes in EUS-TA of solid pancreatic masses.

Most endosonographers worldwide have shifted from FNA needles to FNB needles of 22-G caliber with contemporary end-cutting designs for EUS-TA of solid pancreatic masses. This practice is consistent with recommendations from major professional societies and demonstrates strong widespread guideline adoption [6–8]. There are practice variations in the selection of specific contemporary end-cutting FNB needle designs (Franseen or Fork-tip configurations), which reflects ESGE and ASGE recommendations for contemporary end-cutting needles without endorsement of a specific needle design [6, 7]. When assessing potential sources of variation, geographic location was a factor associated with the choice of needle design, with endosonographers from North America being more likely to use a Fork-Tip needle design while endosonographers from other regions using more commonly a Franseen needle design. Costs and availability of specific FNB needle designs seem to be other reasons that explain global variations, which may be possibly related to partnerships between industry and endoscopy units. As novel needle designs continue emerging, the variations on needle design selection will continue growing and should be monitored to ensure that practices align with research evidence.

Although contemporary FNB needles allow endosonographers to highly accurately diagnose solid pancreatic masses with 2–3 predefined passes [14], most endosonographers prefer a specific method to guide the number of passes. The ESGE and ASGE both recommend against routine use of ROSE when sampling pancreatic masses with FNB needles [6, 7]. Despite these recommendations, over a third of endosonographers still use ROSE during EUS-FNB of pancreatic masses. While the main purpose of ROSE has been to guide the number of passes and improve tissue adequacy, most endosonographers use this service to obtain a preliminary tissue diagnosis that guides conversations with patients and that accelerate the referral process. While the ESGE recommends the use of MOSE, only a fifth of endosonographers use MOSE [7]. Instead of ROSE and MOSE, near two third of endosonographers use VOSE for EUS-FNB of solid pancreatic masses, although this method has not yet been evaluated in RCTs and is not recommended by guidelines. Endosonographers value the published literature and time/efficiency when deciding the method to guide the number of passes, which explains why most endosonographers refrain from using ROSE or MOSE, as both take more time than VOSE without known diagnostic advantages.

It is also noteworthy that only half of endosonographers are monitored on metrics pertaining to diagnostic accuracy, tissue adequacy, or adverse events by their institutions. This practice was much less common in North America compared to other regions of the world. This underscores the need to establish global standards that encourage rigorous monitoring. At the time of data collection for this study, the last guidance on quality indicators on EUS-TA were published in 2015 by the ASGE and American College of Gastroenterology (ACG) and in 2018 by the ESGE [15, 16]. While these guidelines varied slightly, both emphasized thresholds for obtaining a diagnostic sample and detection of malignancy (with the ASGE/ACG guideline also setting standards for adverse events). However, adoption of these previously reported quality indicators appears to lag other common gastrointestinal procedural quality indicators such as the adenoma detection rate (ADR) commonly used among gastroenterologists performing screening and surveillance colonoscopies. One explanation is that compared with ADR [17, 18], quality indicators for EUS-TA for solid pancreatic masses have not been clearly linked to negative patient outcomes. Additionally, setting thresholds for quality indicators may be challenging given the heterogeneity of practices of EUS-TA [19].

Our survey also highlights practice patterns on aspects not emphasized on guidelines due to a limited body of evidence. First, most endosonographers use a fanning technique and stylet slow pull with or without suction when doing EUS-FNB of solid pancreatic masses. One RCT (n = 109) previously found that stylet slow pull provides the best tissue adequacy and diagnostic accuracy with Franseen and Fork-tip needles [20]. One small RCT (n = 54) showed that the fanning technique needs fewer FNA passes compared to the to-and-fro technique for solid pancreatic masses; however, this advantage has not been proven with FNB needles[21, 22]. Second, there is high variation in the number of needle actuations used for each FNB pass, although 60% perform ≤ 10 actuations. Two small RCTs have shown discordant results on this topic, with one study showing that 15 was better than 5 actuations and the other study showing that 3 was similar to 12 actuations [23, 24]. Third, 16% of endosonographers routinely use EUS elastography and 9% use contrast-enhanced EUS. This low adoption may be a consequence of both limited access and possible economic barriers to these technologies as well as the lack of RCT data supporting the use of advanced EUS imaging for tissue acquisition of solid pancreatic masses. Fourth, there was variation in how endosonographers express the tissue out of FNB needles, but it was common to use a stylet, either alone, or followed by an air or saline flush. There are limited data on the impact of different techniques to express tissue with FNB needles, but subtle advantages were noticed with air flushing on reduced blood contamination after FNA sampling [25]. Finally, 80% of endosonographers put the FNB sample on 10% formalin. There are no data comparing one processing technique against others, so this decision has been left to center’s experience and expertise on ESGE guidelines [7].

Our study does have some limitations. As with any survey-based research, responses are self-reported and may not fully reflect actual clinical practice. While a cross-sectional design provides a snapshot of practices during the survey period, it cannot account for temporal changes as new evidence and devices emerge. The survey was distributed only in English, potentially limiting participation from some regions, and we were unable to capture data from Africa, which restricts global generalizability. Although the response rate of 30% is acceptable, non-response bias cannot be excluded, as endosonographers less engaged in guideline-based practice may have been less motivated to participate. Selection bias is also possible as the survey was distributed to endosonographers who were actively engaged with regional societies and/or leaders in EUS-TA, who may be more familiar with guideline recommendations. As a result, our study population was predominantly comprised by a breadth of experienced, high-volume, and academically focused endosonographers. However, subgroup analysis by experience and procedural volume did not influence our results. Despite these limitations, strengths of our study include a large and diverse cohort, broad international representation, and the ability to assess variation across geographic, institutional, and experiential strata.

In conclusion, this large international survey demonstrates that guideline-based practices, particularly the use of FNB needles, have been widely adopted by endosonographers who perform EUS-TA of solid pancreatic masses. However, significant variability persists in sampling techniques, use of advanced imaging modalities, and institutional quality monitoring. These findings highlight opportunities for greater global harmonization of practice standards and the integration of robust quality indicators. Importantly, as technology continues to evolve rapidly in the field of EUS-TA, the data from this survey provide a valuable benchmark to understand current practices and identify areas where innovation is most needed. In this context, surveys such as ours play a critical role, offering timely insights into practice patterns that may be more adaptable than large multicenter trials when technology is changing quickly. By serving as a reference point, this study helps guide the evolution of future tools, techniques, and quality initiatives in EUS-TA of solid pancreatic masses.

## Supplementary Information

Below is the link to the electronic supplementary material.

Supplementary file1 (DOCX 198 KB)
