# Supplementary material for: Practice patterns of endoscopic ultrasound-guided tissue acquisition of solid pancreatic masses: an international survey study
Source: Surg Endosc. 2026 Jul 14;40(10):8460–9. doi: 10.1007/s00464-026-13100-0 (PMC13633374; doi:10.1007/s00464-026-13100-0)
Supplement: Supplementary file 1 [file 464_2026_13100_MOESM1_ESM.docx]

**Supplementary Figure 1.** Influence of preliminary diagnostic interpretation using ROSE on practice patterns.

**Supplementary Table 1.** Variations in practice patterns in EUS-TA of solid pancreatic masses using FNB needles based on endosonographer experience. n (%).

|  | Early Career  (n=184) | Experienced  (n=190) | p-value |
| --- | --- | --- | --- |
| Preferred Needle Type |  |  |  |
| FNB Needle | 165/184 (90) | 172/190 (91) | 0.78 |
| FNA Needle | 17/183 (9) | 18/190 (9) | 0.95 |
| Preferred Needle Size |  |  |  |
| 25-G | 8/178 (4) | 15/182 (8) | 0.03 |
| 22-G | 159/178 (89) | 148/182 (81) | 0.15 |
| Preferred Needle Design^1^ |  |  |  |
| SharkCore (Fork-Tip) | 44/180 (24) | 39/183 (22) | 0.51 |
| Acquire (Franseen) | 77/180 (43) | 78/183 (43) | 0.98 |
| Acquire S (Franseen) | 15/180 (8) | 15/183 (8) | 0.96 |
| SonoTip TopGain (Franseen) | 4/180 (2) | 2/183 (1) | 0.40 |
| EZ Shot 3 Plus (Menghini) | 11/180 (6) | 5/183 (3) | 0.12 |
| EchoTip ProCore (Reverse-bevel) | 3/180 (2) | 6/183 (3) | 0.32 |
| EchoTip AcuCore (Franseen) | 2/180 (1) | 2/183 (1) | 0.99 |
| Trident (Tri-Tip) | 5/180 (3) | 7/183 (4) | 0.58 |
| Needle Selection Criteria^1^ |  |  |  |
| Needle Properties | 2.0 ± 1.2 | 1.7 ± 1.1 | 0.08 |
| Lesion Characteristics | 2.5 ± 1.3 | 2.2 ± 1.3 | 0.02 |
| Data | 2.3 ± 1.4 | 2.4 ± 1.4 | 0.44 |
| Cost | 3.4 ± 1.5 | 3.5 ± 1.4 | 0.46 |
| Availability | 2.7 ± 1.5 | 2.9 ± 1.6 | 0.20 |
| Preffered Needle Movement |  |  |  |
| Fanning | 141/171 (82) | 135/180 (75) | 0.09 |
| To-Fro | 22/171 (13) | 35/180 (19) | 0.10 |
| Initial Sampling Technique |  |  |  |
| Stylet slow-pull alone | 48/170 (28) | 45/180 (25) | 0.50 |
| Stylet slow-pull followed by dry or wet suction | 91/170 (54) | 80/180 (44) | 0.09 |
| Dry suction alone^2^ | 14/170 (8) | 25/180 (14) | 0.09 |
| Plain needle alone without slow-pull or suction | 8/170 (5) | 10/180 (6) | 0.72 |
| Wet section alone^3^ | 4/170 (2) | 15/180 (8) | 0.01 |
| Utilization of Advanced EUS Imaging |  |  |  |
| EUS Elastography | 23/167 (14) | 25/173 (14) | 0.86 |
| Contrast-Enhanced EUS | 11/167 (7) | 15/173 (9) | 0.47 |
| Method to Determine # of Passes |  |  |  |
| VOSE^4^ | 102/167 (61) | 100/170 (59) | 0.67 |
| MOSE^5^ | 35/167 (21) | 37/170 (22) | 0.86 |
| ROSE | 40/167 (24) | 47/170 (28) | 0.44 |
| Predefined | 25/167 (15) | 25/170 (15) | 0.95 |
| Criteria to Determine # of Passes Method^6^ |  |  |  |
| Data | 1.7 ± 1.2 | 2.1 ± 1.4 | <0.01 |
| Time | 2.5 ± 1.3 | 2.7 ± 1.3 | 0.13 |
| Costs | 3.6 ± 1.5 | 3.9 ± 1.3 | 0.13 |
| Availability | 3.4 ± 1.8 | 3.4 ± 1.8 | 0.78 |
| Patient Satisfaction | 3.5 ± 1.6 | 3.6 ± 1.5 | 0.64 |

FNB: fine needle biopsy; FNA: fine needle aspiration; MOSE: macroscopic on-site evaluation; ROSE: rapid on-site cytopathology evaluation.

1. Defined as percentage of respondents who report often or always 2. Defined as applying negative pressure with a syringe. 3. Defined by pre-flushing the needle with saline or sterile water, in addition to applying negative pressure with a syringe. 4. Defined by presence of worm-like whitish or yellowish material. 5. Defined by an aggregated specimen length of macroscopically visible core that measures ~10 mm or more. 6. Responses to 5-point Likert scales were recorded as 1 being the most important and 5 being the least important and treated as continuous variables.

*SharkCore, Medtronic, Minneapolis, USA; Acquire, Boston Scientific, Marlborough, USA; Acquire S, Boston Scientific, Marlborough, USA; SonoTip TopGain, Medi-Globe, Germany; EZ Shot 3 Plus, Olympus, Tokyo, Japan; EchoTip ProCore; Cook Medical, Indiana, USA; EchoTip AcuCore, Cook Medical, Indiana, USA; Trident, Microtech, Nanjing, China.*

**Supplementary Table 2.** Variations in practice patterns in EUS-TA of solid pancreatic masses using FNB needles based on endosonographer practice volume. n (%).

|  | Low Volume  (n=109) | High Volume  (n=263) | p-value |
| --- | --- | --- | --- |
| Preferred Needle Type |  |  |  |
| FNB Needle | 96/109 (88) | 239/263 (91) | 0.41 |
| FNA Needle | 9/108 (8) | 26/263 (10) | 0.64 |
| Preferred Needle Size |  |  |  |
| 25-G | 3/103 (3) | 20/255 (8) | 0.09 |
| 22-G | 85/103 (86) | 220/255 (83) | 0.37 |
| Preferred Needle Design^1^ |  |  |  |
| SharkCore (Fork-Tip) | 22/103 (21) | 61/258 (24) | 0.62 |
| Acquire (Franseen) | 54/103 (52) | 154/258 (60) | 0.21 |
| Acquire S (Franseen) | 4/103 (4) | 26/258 (10) | 0.05 |
| SonoTip TopGain (Franseen) | 3/103 (3) | 3/258 (1) | 0.24 |
| EZ Shot 3 Plus (Menghini) | 8/103 (8) | 8/258 (3) | 0.05 |
| EchoTip ProCore (Reverse-bevel) | 3/103 (3) | 6/258 (2) | 0.75 |
| EchoTip AcuCore (Franseen) | 2/103 (2) | 2/258 (1) | 0.34 |
| Trident (Tri-Tip) | 5/103 (5) | 7/258 (3) | 0.31 |
| Needle Selection Criteria^1^ |  |  |  |
| Needle Properties | 1.8 ± 1.1 | 1.9 ± 1.1 | 0.96 |
| Lesion Characteristics | 2.2 ± 1.3 | 2.4 ± 1.3 | 0.09 |
| Data | 2.3 ± 1.4 | 2.3 ± 1.3 | 0.81 |
| Cost | 3.4 ± 1.5 | 3.5 ± 1.4 | 0.44 |
| Availability | 2.3 ± 1.5 | 3.0 ± 1.6 | <0.001 |
| Preferred Needle Movement |  |  |  |
| Fanning | 82/98 (84) | 194/251 (77) | 0.19 |
| To-Fro | 14/98 (14) | 42/251 (17) | 0.58 |
| Preferred Initial Sampling Technique |  |  |  |
| Stylet slow-pull alone | 36/97 (37) | 58/251 (23) | <0.01 |
| Stylet slow-pull followed by dry or wet suction | 47/97 (48) | 123/251 (49) | 0.93 |
| Dry suction alone^2^ | 6/97 (6) | 32/251 (13) | 0.08 |
| Plain needle alone without slow-pull or suction | 5/97 (5) | 13/251 (5) | 0.99 |
| Wet section alone^3^ | 3/97 (3) | 16/251 (6) | 0.23 |
| Utilization of Advanced EUS Imaging |  |  |  |
| EUS Elastography | 15/91 (16) | 34/247 (14) | 0.53 |
| Contrast-Enhanced EUS | 7/92 (8) | 19/248 (8) | 0.99 |
| Method to Determine # of Passes |  |  |  |
| VOSE^4^ | 51/89 (57) | 149/246 (61) | 0.59 |
| MOSE^5^ | 19/89 (21) | 54/246 (22) | 0.91 |
| ROSE | 20/89 (22) | 66/246 (27) | 0.42 |
| Predefined | 21/89 (24) | 29/246 (12) | <0.01 |
| Criteria to Determine # of Passes Method^6^ |  |  |  |
| Data | 1.8 ± 1.2 | 2.0 ± 1.3 | 0.08 |
| Time | 2.4 ± 1.4 | 2.7 ± 1.3 | 0.02 |
| Costs | 3.6 ± 1.6 | 3.8 ± 1.4 | 0.42 |
| Availability | 3.3 ± 1.9 | 3.4 ± 1.8 | 0.78 |
| Patient Satisfaction | 3.4 ± 1.7 | 3.6 ± 1.5 | 0.42 |

FNB: fine needle biopsy; FNA: fine needle aspiration; MOSE: macroscopic on-site evaluation; ROSE: rapid on-site cytopathology evaluation.

1. Defined as percentage of respondents who report often or always 2. Defined as applying negative pressure with a syringe. 3. Defined by pre-flushing the needle with saline or sterile water, in addition to applying negative pressure with a syringe. 4. Defined by presence of worm-like whitish or yellowish material. 5. Defined by an aggregated specimen length of macroscopically visible core that measures ~10 mm or more. 6. Responses to 5-point Likert scales were recorded as 1 being the most important and 5 being the least important and treated as continuous variables.
*SharkCore, Medtronic, Minneapolis, USA; Acquire, Boston Scientific, Marlborough, USA; Acquire S, Boston Scientific, Marlborough, USA; SonoTip TopGain, Medi-Globe, Germany; EZ Shot 3 Plus, Olympus, Tokyo, Japan; EchoTip ProCore; Cook Medical, Indiana, USA; EchoTip AcuCore, Cook Medical, Indiana, USA; Trident, Microtech, Nanjing, China.*
